# Oral Administration of Bovine Milk-Derived Extracellular Vesicles Attenuates Cartilage Degeneration via Modulating Gut Microbiota in DMM-Induced Mice

**DOI:** 10.3390/nu15030747

**Published:** 2023-02-01

**Authors:** Qiqi Liu, Haining Hao, Jiankun Li, Ting Zheng, Yukun Yao, Xiaoying Tian, Zhe Zhang, Huaxi Yi

**Affiliations:** 1 College of Food Science and Engineering, Ocean University of China, Qingdao 266000, China; 2 Food Laboratory of Zhongyuan, Luohe 462300, China

**Keywords:** bovine milk, extracellular vesicles, osteoarthritis, cartilage degeneration, gut microbiota

## Abstract

Osteoarthritis (OA) is the most common joint disease primarily characterized by cartilage degeneration. Milk-derived extracellular vesicles (mEVs) were reported to inhibit catabolic and inflammatory processes in the cartilage of OA patients. However, the current therapies target the advanced symptoms of OA, and it is significant to develop a novel strategy to inhibit the processes driving OA pathology. In this study, we investigated the therapeutic potential of mEVs in alleviating OA in vivo. The results revealed that mEVs ameliorated cartilage degeneration by increasing hyaline cartilage thickness, decreasing histological Osteoarthritis Research Society International (OARSI) scores, enhancing matrix synthesis, and reducing the expression of cartilage destructive enzymes in the destabilization of medial meniscus (DMM) mice. In addition, the disturbed gut microbiota in DMM mice was partially improved upon treatment with mEVs. It was observed that the pro-inflammatory bacteria (*Proteobacteria*) were reduced and the potential beneficial bacteria (*Firmicutes*, *Ruminococcaceae*, *Akkermansiaceae*) were increased. mEVs could alleviate the progression of OA by restoring matrix homeostasis and reshaping the gut microbiota. These findings suggested that mEVs might be a potential therapeutic dietary supplement for the treatment of OA.

## 1. Introduction

Osteoarthritis (OA) is one of the most common musculoskeletal diseases characterized by cartilage degeneration [1]. Due to global aging and the increase in obesity, the OA burden poses a serious challenge to healthy aging. OA affects approximately 303.1 million people worldwide and has become the common cause of disability [2]. Currently, there are no approved disease-modifying drugs available, and the main therapies for OA are typically focused on pain control, intra-articular injections, and joint replacement, all of which simply target the symptoms of advanced disease [3,4]. Therefore, it is significant to develop effective and safe strategies to prevent or treat OA.

Milk-derived extracellular vesicles (mEVs) are nanoparticles and carry a cargo rich in proteins, lipids, and microRNAs that can be transported between cells [5,6]. It has been reported that mEVs could provide protection to the luminal cargo against harsh degrading conditions of the gut and were subsequently detected in multiple organs [6,7,8]. In recent years, there is an emerging interest in the use of mEVs as biomarkers or treatments for various diseases, such as breast cancer [6], ulcerative colitis [7], and rheumatoid arthritis [9]. However, only one study has shown that TGF-β and miR-148a, which are carried by mEVs, have the potential for cartilage protection in vitro, but the underlying mechanism of mEVs alleviating cartilage degeneration remains to be explored [10].

Gut microbiota has been regarded as an important factor in the development of OA [11,12]. There is increasing interest in the relationship between gut microbiota and OA. Alteration in normal gut microbiota has been implicated in metabolic syndrome and inflammation, which are also important components in the development of OA [13]. A recent work has suggested the role of gut microbiota in OA. For instance, the destabilization of the medial meniscus (DMM)-induced mice conventionally had more severe joint damage compared with the germ-free mice [14]. Several groups have reported that intervention of supplementation with either probiotics or prebiotics decreased the histological changes associated with OA and modified systemic inflammation profiles [15,16,17]. mEVs have been shown to regulate intestinal immunity by altering gut microbiota in both healthy and diseased mice [7,18]. In this study, the therapeutic effects of mEVs on cartilage degeneration and gut microbiota were investigated in the DMM-induced OA mice model.

## 2. Materials and Methods

### 2.1. Isolation and Identification of mEVs

Bovine milk contains a large number of non-EV proteins, milk fat, sugars, and other components. To isolate and purify mEVs, we developed an efficient method in our previous study [18,19]. Raw milk was produced from lactating Holstein cows obtained from a local dairy farm, and the samples were immediately transported under refrigeration to the laboratory within 2 h. The raw milk was incubated with 0.05% chymosin and 0.3% CaCl_2_ at 4 °C for 90 min. To remove milk fat globules, residual chymosin, and other debris, the sample was centrifuged at 16,500× *g* for 30 min at 4 °C. The supernatant was filtered by 0.22 μm filters (Millipore, Hayward, CA, USA) and ultracentrifuged at 100,000× *g* at 4 °C for 1 h (Hitachi, Ltd., Tokyo, Japan). After being filtered by 0.22 μm filters, the supernatant was ultracentrifuged at 135,000× *g* at 4 °C for 90 min. The pellets were resuspended with PBS and washed by ultracentrifugation at 100,000× *g* at 4 °C for 1 h. The mEVs were resuspended in PBS and transferred onto the 100 kDa filters (Millipore, Hayward, CA, USA) through 0.22 μm filters and centrifuged at 5000× *g* for 30 min twice. The mEVs were resuspended in PBS, and the protein concentration was determined using the BCA kit (Beyotime, Shanghai, China). The mEVs were stored at 4 °C for 1 week.

The size distribution and concentration were detected by nanoparticle tracking analysis (NTA) using ZetaView PMX 100 (Particle Metrix, Meerbusch, Germany). The size distribution and particle concentration were evaluated in three replicates. Transmission electron microscopy (TEM) was employed to observe the morphology of mEVs, and mEVs were imaged using HITACHI HT7700 Transmission Electron Microscope (Hitachi, Ltd., Tokyo, Japan). The mEV marker proteins (TSG101, CD81, CD63) and negative control (calnexin) were analyzed by Western blotting. Primary antibodies against TSG101 (ab133586), Calnexin (ab227310), and CD63 (ab134045) were purchased from Abcam (Cambridge, UK). CD81 (GTX101766) was purchased from GeneTex (Irvine, CA, USA).

### 2.2. Animal Experiments

A total of 57 specific pathogen-free male C57BL/6J mice (7 weeks of age) were purchased from Beijing Vita River Laboratory Animal Technology Co., Ltd. (Beijing, China). All animal experiments were in accordance with the Committee on the Ethics of Animal Experiments of Ocean University of China (permission number: spxy2021082701). Mice were housed at 22 °C with 12 h light/dark cycles. After a 1-week adaption period, mice were divided into three groups randomly: sham group (PBS), DMM + PBS group (PBS), DMM + mEVs group (mEVs, 0.2 mg/kg of body weight). Then, all groups were gavaged once daily with PBS or mEVs and kept for 12 weeks.

After 2 weeks of gavage, OA was induced by surgical destabilization of the medial meniscus (DMM) of the right knee in DMM + PBS and DMM + mEVs groups. Specifically, mice were anesthetized with 5% chloral hydrate by intraperitoneal injection, then the joint capsule was opened, and the medial meniscotibial ligament was cut to destabilize the meniscus. In the sham group, the joint capsule was surgically opened, but the medial meniscotibial ligament was intact. At 10 weeks after surgery, the mice were anesthetized, and blood was taken from the eyeballs. The serum was obtained by centrifugation at 4000× *g* for 40 min at 4 °C. The levels of cartilage degradation markers C-telopeptide of type Ⅱ collagen (CTX-Ⅱ) and cartilage oligomeric matrix protein (COMP) in the serum were detected using enzyme-linked immunosorbent assay (ELISA) kits according to the manufacturer’s instructions (Nanjing Jiancheng Bio, Nanjing, China).

### 2.3. Histology and Immunohistochemistry

The right knee joints were fixed with 4% paraformaldehyde, decalcified with 10% EDTA for 4 weeks, and then embedded in paraffin. The sections (3 μm in thickness) were cut and then stained with hematoxylin–eosin (H&E), safranin o-fast green (Safranin O), and toluidine blue. The cartilage thickness in the tibial plateau, including hyaline cartilage (HC) and calcified cartilage (CC), was measured with HE staining and determined by Image J software (National Institutes of Health, Bethesda, MD, USA). Distances between surfaces of articular cartilage and the tidemark line indicate hyaline cartilage thickness, and distances between the tidemark line and subchondral bone plate (SBP) indicate calcified cartilage thickness. The Osteoarthritis Research Society International (OARSI) scores were used to grade the cartilage degeneration of the knee joint [20]. For immunofluorescence, antigens were retrieved by boiling tissue section in sodium citrate buffer, and endogenous peroxidase activity was blocked by incubation in a 3% hydrogen peroxide solution. The tissue sections were incubated with 3% BSA for 30 min at room temperature to block non-specific binding, followed by straining with the primary antibodies overnight at 4 °C. After the secondary antibody incubation, the sections were stained with DAB and hematoxylin and visualized under a light microscope (Olympus, Tokyo, Japan).

### 2.4. 16S rRNA Bacterial Sequencing

Mice feces were obtained 10 weeks after surgery. Fecal pellets were freshly harvested from mice and immediately frozen in liquid nitrogen and stored at −80 °C. The genomic DNA of feces was extracted, and the bacterial 16S rRNA genes V3–V4 region was amplified using the forward primer 338F (5′-ACTCCTACGGGAGGCAGCA-3′) and the reverse primer 806R (5′-GGACTACHVGGGTWTCTAAT-3′). Sample-specific 7 bp barcodes were incorporated into the primers for multiplex sequencing. PCR amplicons were purified, quantified, and pooled in equal amounts. The paired-end 2 × 250 bp sequencing was performed using the Illumina MiSeq platform with MiSeq Reagent Kit V3 at Shanghai Personal Biotechnology Co., Ltd. (Shanghai, China). For alpha diversity analysis, the indexes of Chao1, Observed_species, Shannon, Simpson, Faith_pd, and Pielou_e were calculated using QIIME2, R software and ggplot2 package. For analyzing beta diversity, principal coordinate analysis was performed via QIIME2, R software and ape package. Linear discriminant analysis Effect Size (LEfSe) was performed to detect differentially abundant taxa across groups using Python LEfSe package, R software and ggtree package.

### 2.5. Statistical Analysis

All results were expressed as mean ± standard deviations (SD). One-way analysis of variance (ANOVA) with Tukey’s test for multiple comparisons was performed using GraphPad Prime 8 (GraphPad Software, San Diego, CA, USA). A *p* value less than 0.05 was considered to be statistically significant. All analyses and graphing of the data were carried out using GraphPad Prime 8.

## 3. Results

### 3.1. Characterization of mEVs

**Figure 1:**
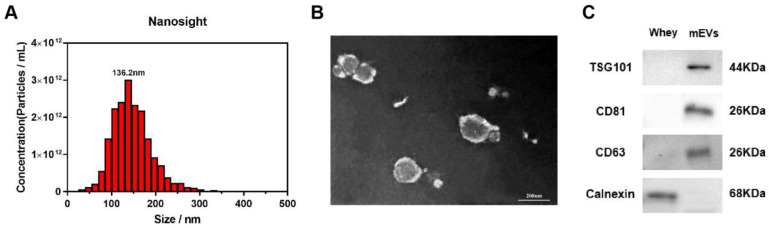
Characterization of mEVs. (**A**) The size distribution of mEVs was measured by NTA (Nanoparticle tracking analysis). The peak diameter was about 136.2 nm. (**B**) Transmission electron microscopy images of mEVs. Scale bars, 200 nm. (**C**) mEV markers and calnexin were assessed by Western blot analysis.

The isolated mEVs were characterized by NTA, TEM, and Western blot. The results demonstrated that the diameters of the isolated mEVs were distributed among 100–200 nm, and the mean peak of particle size was located at 136.2 nm (Figure 1A). The image in Figure 1B showed that the isolated mEVs were typically round spherical shapes with double-layer membranes. Moreover, the EV-positive markers TSG101, CD81, and CD63 were expressed in isolated mEVs, and Calnexin was not detected (Figure 1C).

### 3.2. mEVs Reverse Cartilage Degeneration in DMM-Induced Mice

**Figure 2:**
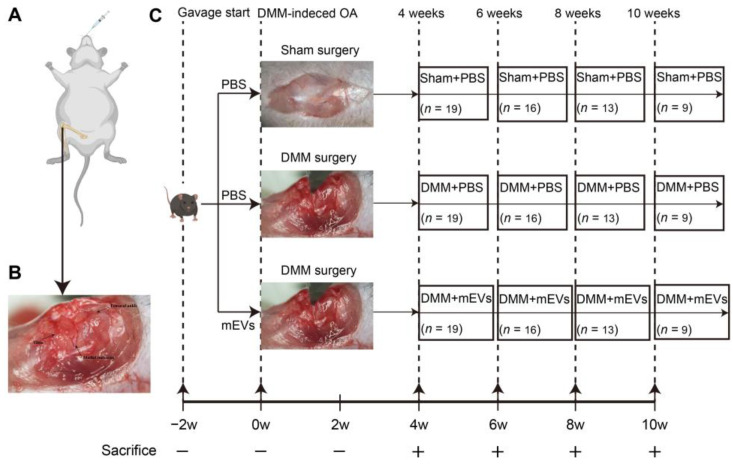
Schematic illustration of in vivo experimental design. (**A**) Schematic diagram of animal model showing DMM-induced molded in the right hind limb and treated by oral administration. (**B**) Position of the femur, tibia, and medial meniscus in the knee joint. (**C**) Mice were pre-gavaged for 2 weeks, and then, DMM surgery was performed to establish the OA model, and tissue samples were harvested every 2 weeks from the fourth week after surgery for subsequent analysis. The number of samples in each group is expressed as an n value.

In this study, mice were gavaged with PBS or mEVs for 2 weeks before DMM surgery. To assess the therapeutic effect of mEVs in the development of OA, knee joints from each group of animals at 4, 6, 8 and 10 weeks post-surgery were harvested for histological analysis (Figure 2). To determine the severity of DMM-induced OA, the serum levels of CTX-Ⅱ and COMP were measured at different stages. The results showed that the serum CTX-Ⅱ and COMP in the DMM+PBS group were significantly higher than those of the other two groups at 8 weeks post-surgery, indicating that the OA model was successfully established and mEVs could effectively prevent the progression of OA (Supplementary Material Figures S1 and S2).

**Figure 3:**
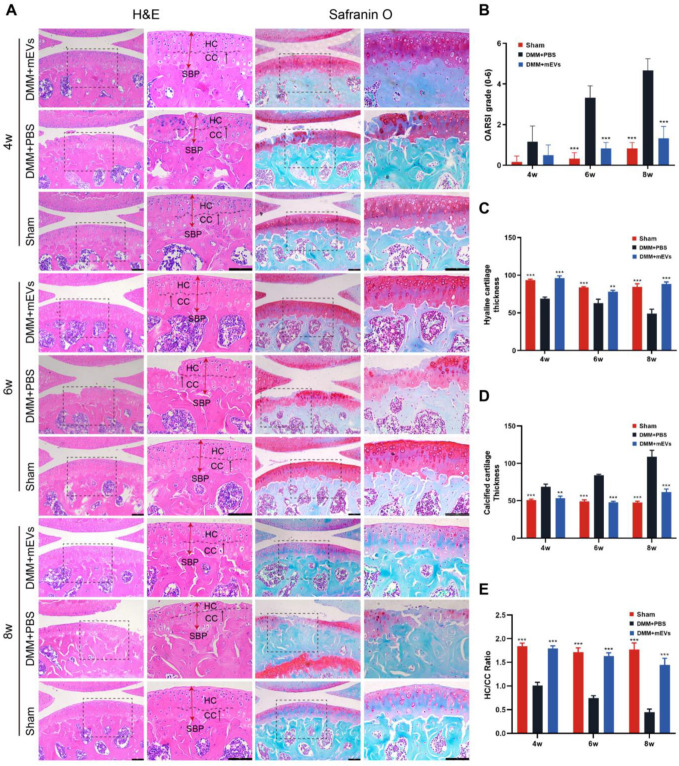
Effects of mEVs on articular cartilage structure in DMM-induced mice. (**A**) Hematoxylin–eosin and safranin o-fast green staining at 4, 6, and 8 weeks after DMM surgery. Scale bars: 100 μm. The tibial plateau cartilage thickness in the weight-bearing area is indicated by red double-headed arrows. The black arrow indicates the tidemark (boundary of HC and CC) of cartilage. HC, hyaline cartilage; CC, calcified cartilage; SBP, subchondral bone plate. (**B**) The joint lesions were graded on a scale of 0–6 using the OARSI scoring system. Data were presented as the mean ± SD (*n* = 5). (**C**–**E**) Quantification of cartilage thickness of all groups. All data are shown as means ± SD deviation (*n* = 3). ** p < 0.01, *** p < 0.001 vs. DMM+PBS group.

Hematoxylin–eosin and safranin o-fast green staining showed the occurrence of cartilage degeneration and cartilage proteoglycan loss after DMM surgery, and the OARSI scoring was taken to quantify the severity of cartilage damage (Figure 3 and Supplementary Material Table S1). At the early time point of 4 weeks after surgery, there were no significant differences in OARSI scores among the three groups, but the articular cartilage was locally damaged in the DMM+PBS group. Compared to the DMM+PBS group, the ratio of HC/CC was increased by 77.94% in the DMM+mEVs group. At 6 weeks post-surgery, the DMM group showed partial loss of hyaline cartilage and a significant increase in OARSI score, while the HC/CC ratio was significantly increased by 118.67% in the DMM+mEVs group in comparison to the DMM+PBS group. At 8 weeks post-surgery, the DMM+PBS group showed a significant decrease in hyaline cartilage thickness, a marked increase in OARSI scores, severe cartilage erosion, and exposure of subchondral bone. However, mEVs treatment protected against cartilage degeneration, and the HC/CC ratio increased by 219.92% compared to that of the DMM+PBS group. As described above, the articular cartilage showed local wear at 4 weeks post-surgery, and the severe cartilage tissue erosion and exposure of subchondral bone could be observed at 8 weeks post-surgery. mEVs treatment could reduce cartilage tissue damage and attenuate the development of cartilage erosion after DMM surgery.

**Figure 4:**
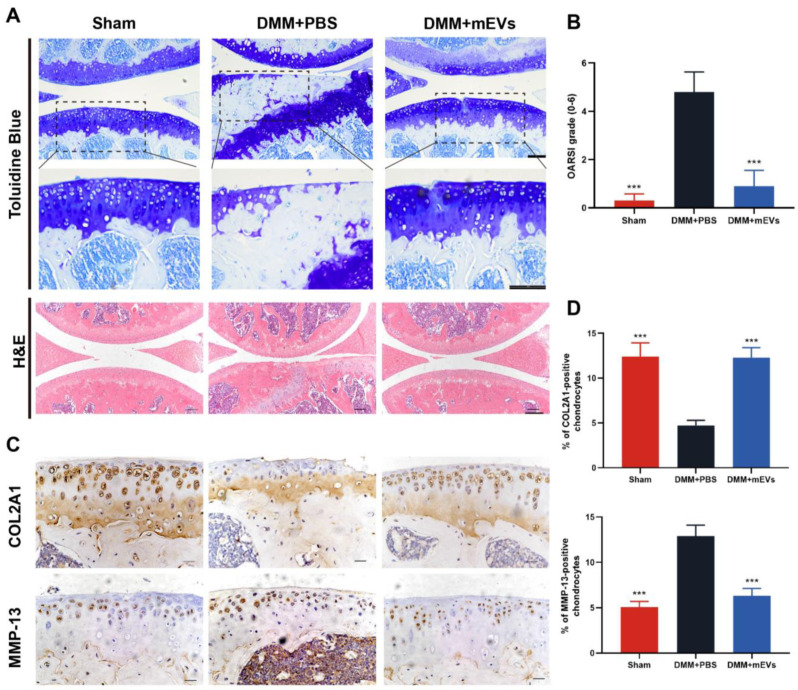
Effects of mEVs on cartilage degradation in DMM-induced mice. (**A**) Toluidine blue and hematoxylin–eosin-stained sections of knee joints. Scale bars: 100 μm. The dashed black box indicates the magnified region. (**B**) The joint lesions were graded on a scale of 0–6 using the OARSI scoring system. Data were presented as the mean ± SD (*n* = 5). (**C**,**D**) Immunohistochemistry analysis of COL2A1 and MMP-13 in sections of tibial plateau of knee joints. Scale bar, 25 μm. All data are shown as means ± SD deviation (*n* = 5). *** p < 0.001 vs. DMM+PBS group.

Toluidine blue and hematoxylin–eosin staining showed cartilage degeneration at 10 weeks post-surgery (Figure 4). The DMM+PBS group displayed severely calcified cartilage and extensive loss of cartilage, whereas the mEVs treatment groups showed more complete integration of cartilage with a smooth surface (Figure 4A). The OARSI scoring was taken to quantify the severity of cartilage damage, and the result showed that the mEVs treatment group significantly relieved the cartilage lesion compared with the DMM+PBS group. To further examine the effect of mEVs on cartilage degeneration, an immunohistochemical experiment was conducted to assess the change in COL2A1 and MMP-13 protein levels in articular cartilage. As shown in Figure 4C,D, the DMM-induced decrease in COL2A1 could be largely reversed by mEVs. The mEVs treatment strongly reduced the percentage of MMP-13-positive chondrocytes in DMM-induced mice compared with that of the DMM+PBS group. These results indicated that mEVs treatment in DMM-induced mice promoted chondrocyte anabolism and inhibited its catabolism effectively, which prevented erosion of the cartilage matrix after DMM surgery.

### 3.3. mEVs Rectify Gut Microbiota Imbalance in DMM-Induced Mice

**Figure 5:**
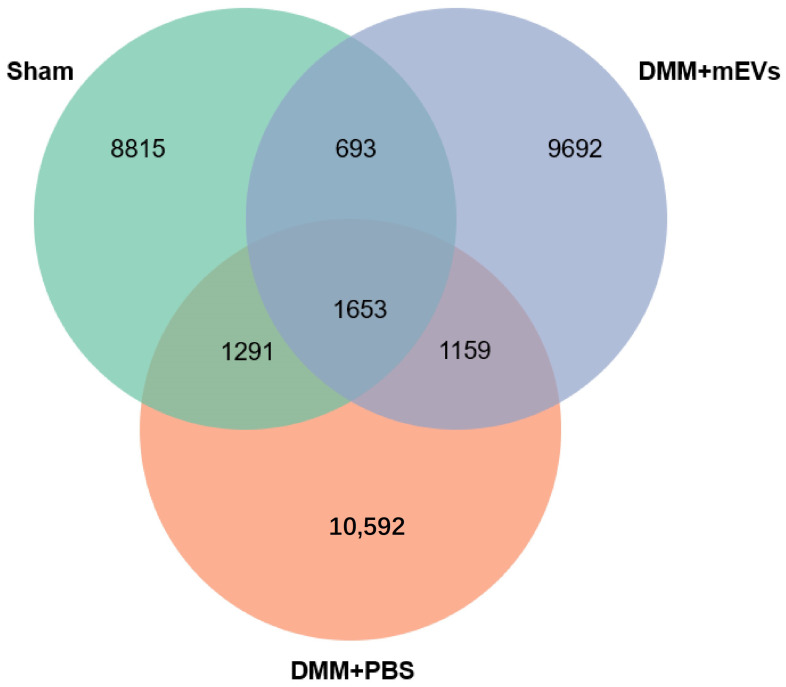
Venn diagram of ASV/OUT in the feces. The numbers of ASVs/OTUs were evaluated by 16S rRNA gene V3–V4 pyrosequencing reads. ASVs, amplicon sequence variants; OTUs, operational taxonomic units.

**Figure 6:**
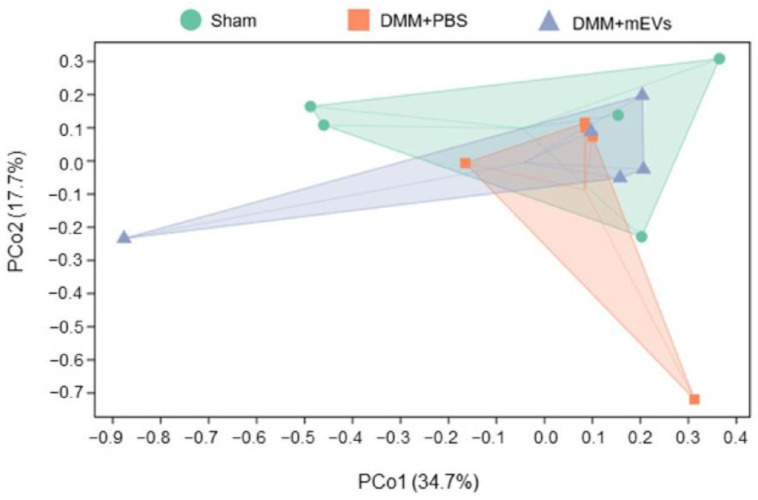
β-diversity evaluated using weighted_unifrac based PCoA (*n* = 5).

To characterize the gut microbiota in DMM-induced mice under different treatments, microbiota in fecal samples were analyzed from three groups of mice (sham group, DMM+PBS group, DMM+mEVs group) by 16S rRNA sequencing. The results showed that the sham group and DMM+PBS group had distinct intestinal microbial populations, and mEVs tended to reverse the key changes induced by DMM surgery. Specifically, a higher number of ASVs/OTU was observed in DMM-induced mice compared with that of sham-operated or mEVs-treated mice (Figure 5). As reflected by the indexes of Chao 1, Observed_species, Shannon, Simpson, Faith_pd, and Pielou_e, the microbiota in DMM-induced mice showed a higher α-diversity compared with that of the sham group and DMM+mEVs group, but there were no significant differences (*p* > 0.05) (Table 1). Furthermore, β-diversity was observed by the principle coordinate analysis (PCoA). As shown in Figure 6, PCoA revealed that the sham group and the DMM+mEVs group had more similar microbial community, but the differences among the three groups were not significant. These results indicated that mEVs treatment decreased the diversity of gut microbiota and thereby reversed the dysregulated microbiota community structure of DMM-induced mice.

**Figure 7:**
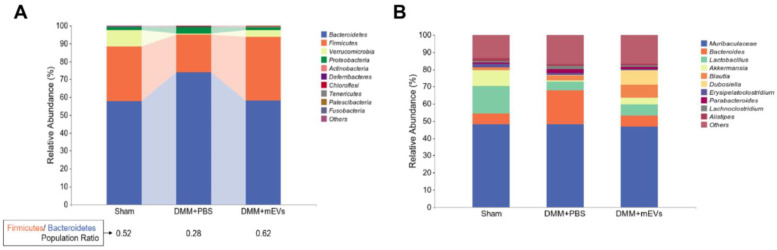
The relative abundance of phylum/genus in each experimental group. (**A**) The relative abundance of phylum in each experimental group. The ratio of the relative abundance of *Firmicutes* to *Bacteroidetes* for each group was calculated. (**B**) The relative abundance of genus in each experimental group.

To further investigate the changes in intestinal flora composition in DMM-induced mice with or without mEVs treatment, taxonomic composition analysis was used to determine the differences in bacterial abundance at different levels. We first observed the changes in bacterial abundance in different treatment groups at the phylum and genus levels. It has been reported that OA patients have a higher relative abundance of *Bacteroides* and a lower relative abundance of *Firmicutes* compared to healthy people [19]. As shown in Figure 7A, it was observed that *Bacteroidetes* and *Firmicutes* were the main phylum in mice, *Bacteroidetes* were increased and *Firmicutes* were decreased in DMM-induced mice, whereas mEVs treatment reversed those changes. Furthermore, mEVs treatment increased the *Firmicutes*/*Bacteroidetes* (*F*/*B*) ratio in DMM-induced mice. DMM surgery resulted in an increase in *Proteobacteria* and a decrease in *Firmicutes* at the phylum. At the genus level, the relative abundance of *Bacteroides* was highest in DMM+PBS group among three groups, while the DMM+PBS group had the lowest relative abundance of *Lactobacillus* and *Akkermansia* among the three groups, but there were no significant differences in relative abundance of *Muribaculaceae* among the three groups (Figure 7B).

At the family and genus level, the ratio of certain flora was also altered in DMM-induced mice. The relative abundance of *Ruminococcaceae* and *Akkermansiaceae* was decreased, and *Bacteroidaceae* and *Enterobacteriaceae* were increased at the family level in DMM-induced mice. Additionally, it has been demonstrated that the abundance of *Enterobacter* increased as arthritis progressed in CIA-susceptible mice [21]. In DMM-induced mice, *Enterobacteriaceae* was observed to be richer compared to the sham group and DMM+mEVs group. At the genus level, DMM-induced mice displayed a reduction of *Lactobacillus*, *Akkermansia*, and *Lachnospiraceae_NK4A136_group*, and the abundance of *Bacteroides* increased in DMM-induced mice, which were partially reversed by mEVs treatment (Table 2). In addition, *Akkermansia_muciniphila* and *Muribaculaceae_mouse_gut* decreased in the feces of DMM-induced mice (Supplementary Material Figure S3).

**Figure 8:**
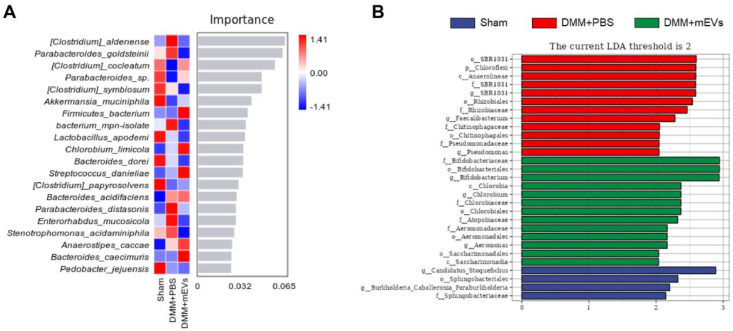
Specific species were altered in each experimental group. (**A**) Bar plot species importance at the species level performed by random forest analysis. (**B**) Differentially enriched gut microbiota in each group of mice at the species level by linear discriminant analysis (LDA).

In addition, random forest analysis and line discriminant analysis (LDA) can detect species that differ significantly in abundance among groups (Figure 8). Random forest analysis was applied to screen out the indicative species. It was observed that *Clostridium_aldenense*, *Parabacteroides_goldsteinii*, *Clostridium_cocleatum* and *Parabacteroides_sp.* had high importance that differed in abundance among groups. *Clostridium_aldenense* and *Parabacteroides_goldsteinii* were more abundant in the DMM+PBS group, while *Clostridium_cocleatum* and *Parabacteroides_sp.* were more abundant in the sham group and DMM+mEVs group (Figure 8A). LDA analysis revealed that key phylotypes were determined as distinguished biomarkers. It was found that some bacteria were overrepresented in the DMM+PBS group, such as *SBR1031* order, *Chloroflexi* phylum, and *Anaerolineae* class, which might be the potential biomarkers and intervention targets of OA therapy (Figure 8B and Figure S4). Furthermore, *Bifidobacteriaceae* family, *Bifidobacteriales* order, and *Bifidobacterium* genus were overrepresented in the DMM+mEVs group, and *Candidatus_Stoquefichus* genus was overrepresented in the sham group (Figure 8B).

## 4. Discussion

In the present study, we investigated the therapeutic effects of mEVs on OA in DMM-induced mice. EVs can be considered regulators of cell communication to transfer bioactive cargos (lipids, miRNAs, proteins) to elicit diverse cellular responses in recipient cells [22]. To date, mEVs have been used in many diseases, but only one study has reported its protection on cartilage from OA patients. It was demonstrated that mEVs had a potential therapy on human osteoarthritic joints by reducing the expression of cartilage-destructive enzymes and pro-inflammatory cytokines in vitro [10]. In our initial observation, it was found that oral administration of mEVs alleviated the progress of cartilage degeneration effectively in the DMM-induced OA model. In addition, a single dose of mEVs was used to interfere with DMM-induced mice in this study; more doses should be selected to fully elucidate the inhibitory effect of mEVs on cartilage degeneration in subsequent studies.

The surgical model of DMM was used to explore the effect of mEVs on OA in vivo. Most notably, it is the first study, to the best of our knowledge, to evaluate the prevention effect of mEVs in the DMM-induced OA model. One of the important findings in our study is that oral administration of mEVs prevents the progress of cartilage degeneration effectively in the DMM-induced OA model. Hematoxylin–eosin, safranin o-fast green and toluidine blue staining revealed that mEVs significantly inhibited the extracellular matrix loss and improved cartilage destruction. By the end of the 12-week treatment, mEV-treated joints showed remarkable restoration of cartilage structure destruction and cartilage matrix degradation that were comparable to that of the sham group.

mEVs not only protect cartilage from damage, but also reshape and restore the composition of gut microbiota in DMM-induced mice. The microbiota is a potential driver of chronic innate activation in OA, but few studies have examined gut microbiota perturbations in OA. In a population-based cohort, OA was found to be associated with a large number of gut microbiota features, and the abundance of gut microbes was negatively associated with the prevalence of OA [23]. A recent paper reported that four bacterial clades were associated with knee pain, including *Bacilli* class, *Lactobacillales* order, *Streptococcaceae* family, and *Streptococcus* genus [24]. Furthermore, the abundant diversity of bacterial nucleic acids has been identified in the synovial fluid and cartilage samples from knee OA patients, such as *Lanchospiraeae* family and *Coriobacteriales* order [25,26]. These evidences indicated that gut microbiota might influence joint biology in OA. In line with previous reports, our findings suggested that the composition of gut microbiota was disturbed in the DMM-induced OA model, but mEVs could inhibit the development of OA by reshaping gut microbiota.

There was an interesting finding that the DMM+PBS group showed a higher α-diversity compared with that of the sham group and DMM+mEVs group. Similarly, the number of observed species was slightly higher in the bacteriome of OA patients compared with that in healthy controls via whole-metagenome shotgun sequencing, but there were no significant differences [27]. Another research study also reported that α-diversity was higher in the OA rats than that in control rats [28]. The mechanism of this phenomenon needs to be further explored. The DMM+PBS group had higher relative abundance of *Proteobacteria* and lower relative abundance of *Firmicutes* and *Ruminococcaceae* compared to the other groups. It has been reported that the enrichment of *Proteobacteria* or decrease in *Firmicutes* is associated with the plasma levels of pro-inflammatory cytokines (e.g., IL-6, IL-8) [29], and *Ruminococcaceae* has been proven to be negatively correlated with the OARSI scores and pro-inflammatory factors (e.g., IL-6, IL-1β) [30]. Moreover, the *F/B* ratio of DMM+PBS group was the lowest among three groups, while The *F/B* ratio has been considered to be associated with maintaining normal intestinal homeostasis, and changes in this ratio can lead to a variety of pathologies, such as obesity and bowel inflammation [31]. *Lactobacillus* has been reported to be mainly enriched in OA monkeys [32]. In contrast, in this study, *Lactobacillus* decreased in the feces of DMM-induced mice. Collectively, these results suggested that oral gavage of mEVs could restore the taxonomic imbalance of gut microbiota induced by DMM surgery. However, there were no significant differences among the three groups, likely due to small group sizes, and the exact mechanisms need further study.

There was some emerging evidence suggesting the correlation between microbiome and the pathogenesis of OA, and the ‘two-hit’ model of OA pathogenesis and enhancement was proposed. One ’hit’ was provided by activation of the adverse gut microbiome of innate immunity, and the other ’hit’ was underlying joint damage [33]. It emphasized that the initiation and perpetuation of OA were associated with innate immune activity, including macrophage-associated inflammatory responses, TLR activation, and complement activation [34,35,36]. TLR2 and TLR4, upregulated in OA cartilage, are stimulated by lipopolysaccharide (LPS) from an outer membrane component of Gram-negative bacteria [37]. In human OA cartilage, an increased fraction of Gram-negative constituent bacterial DNA was detected as well as increased Kyoto Encyclopedia of Gene and Genomes (KEGG) pathways associated with LPS biosynthesis in OA [26]. *Bacteroidetes* are a phylum of Gram-negative bacteria that impact digestion, immunity, and resistance to infection [38]. In agreement with these studies, our data demonstrated an increased abundance of *Bacteroidetes* in the DMM+PBS group, while oral administration of mEVs restored its abundance to a normal level. The abundance of *Enterobacteriaceae* (Gram-negative bacteria) increased in DMM-induced mice, which is positively correlated with the progression of arthritis [21]. Based on the above, we hypothesize that exacerbation of OA may be associated with increased LPS produced by abundant Gram-negative bacteria. In this hypothesis, mEVs may inhibit the production of LPS by altering the composition of gut microbiota. It has not been tested in our study and needs to be explored in further experiments.

Moreover, oral administration of mEVs upregulated the abundance of some beneficial gut microbes, such as *Ruminococcaceae*, *Lachnospiraceae_NK4A136_group*, and *Akkermansia_muciniphila* (*A. muciniphila*). It has been demonstrated that *Ruminococcaceae* was negatively correlated with the OARSI scores, and we found that the DMM+PBS group had a lower abundance of *Ruminococcaceae* with higher OARSI scores. *A. muciniphila* is a promising probiotic that can stimulate mucosal immunity to prevent pathogenic invasion and promote the thickening of the colonic mucosa [39]. The results showed that the special bacteria were affected by mEVs, but how mEVs affect OA progression via regulating gut microbial metabolism remains to be elucidated.

At present, few studies have focused on the effect of mEVs on OA. Our research initially demonstrates that oral administration of mEVs can inhibit cartilage degeneration and restore disordered gut microbiota in DMM-induced mice, but the mechanism needs to be further investigated. Meanwhile, mEV contains abundant functional cargo, such as microRNAs and proteins. It is necessary to explore which substances play the main role in alleviating OA.

## 5. Conclusions

This was the first report that mEVs can attenuate cartilage degeneration by suppressing the expression of catabolism, enhancing matrix synthesis, and reshaping the gut microbiota in DMM-induced OA mice. Oral administration of mEVs might be a potential approach in the treatment of OA.

## Figures and Tables

**Table 1:** Alpha diversity indexes were calculated with QIME2 according to AVS/OUT numbers of each group (*n* = 5).

Alpha Diversity Indexes/Groups	Sham	DMM+PBS	DMM+mEVs
Chao1	3460.11 ± 908.70	4081.75 ± 1749.60	3319.48 ± 1632.58
Observed_species	3080.24 ± 75.15	3747.52 ± 1571.90	3065.48 ± 1635.47
Shannon	7.64 ± 0.38	8.64 ± 1.79	7.64 ± 2.33
Simpson	0.94 ± 0.03	0.96 ± 0.06	0.93 ± 0.09
Faith_pd	179.63 ± 36.40	190.00 ± 63.78	180.54 ± 54.26
Pielou_e	0.66 ± 0.03	0.73 ± 0.10	0.66± 0.16

Data are presented as the mean ± SD (*n* = 5).

**Table 2:** Relative abundance of several bacteria at the family/genus level (*n* = 5).

Family/Genus	Strains	Sham (%)	DMM+PBS (%)	DMM+mEVs (%)
Family	Bacteroidaceae	0.06 ± 0.07	0.20 ± 0.29	0.06 ± 0.04
	Ruminococcaceae	0.03 ± 0.03	0.02 ± 0.01	0.03 ± 0.03
	Akkermansiaceae	0.09 ± 0.16	0.01 ± 0.01	0.04 ± 0.06
	Enterobacteriaceae	0.00 ± 0.00	0.02 ± 0.04	0.00 ± 0.01
Genus	Bacteroides	0.06 ± 0.07	0.20 ± 0.29	0.06 ± 0.04
	Lactobacillus	0.16 ± 0.21	0.05 ± 0.06	0.07 ± 0.12
	Akkermansia	0.09 ± 0.16	0.01 ± 0.01	0.04 ± 0.06
	Lachnospiraceae_NK4A136_group	0.02 ± 0.03	0.00 ± 0.00	0.02 ± 0.02

Data are presented as the mean ± SD (*n* = 5).

## Data Availability

The data presented in this study are available on request from the corresponding author.

## Supplementary Materials

The following supporting information can be downloaded at: https://www.mdpi.com/article/10.3390/nu15030747/s1, Table S1: The OARSI scoring system; Figure S1: The serum CTX-Ⅱ levels were determined by ELISA (*n* = 3); Figure S2: The serum COMP levels were determined by ELISA (*n* = 3); Figure S3: The relative abundance of species in each experimental group; Figure S4: Cladogram based on linear discriminant analysis effect size (LEfSe) analysis showing community composition of the gut microbiota in mice.
