# Supplementary material for: Oral Administration of Bovine Milk-Derived Extracellular Vesicles Attenuates Cartilage Degeneration via Modulating Gut Microbiota in DMM-Induced Mice
Source: Nutrients. 2023 Feb 1;15(3):747. doi: 10.3390/nu15030747 (PMC9920331; doi:10.3390/nu15030747)
Supplement: Supplementary file 1 [file nutrients-15-00747-s001.zip › nutrients-2182990-supplementary.pdf]

Table S1 The OARSI scoring system.

	Score
Normal	0
Loss of Safranin O without structural changes	0.5
Small fibrillations without loss of cartilage	1
Vertical clefts down to the layer immediately below the superficial layer and some loss of surface lamina	2
Vertical clefts/erosion to the calcified cartilage extending to <25% of the articular surface	3
Vertical clefts/erosion to the calcified cartilage extending to 25-50% of the articular surface	4
Vertical clefts/erosion to the calcified cartilage extending to 50-75% of the articular surface	5
Vertical clefts/erosion to the calcified cartilage extending to >75% of the articular surface	6

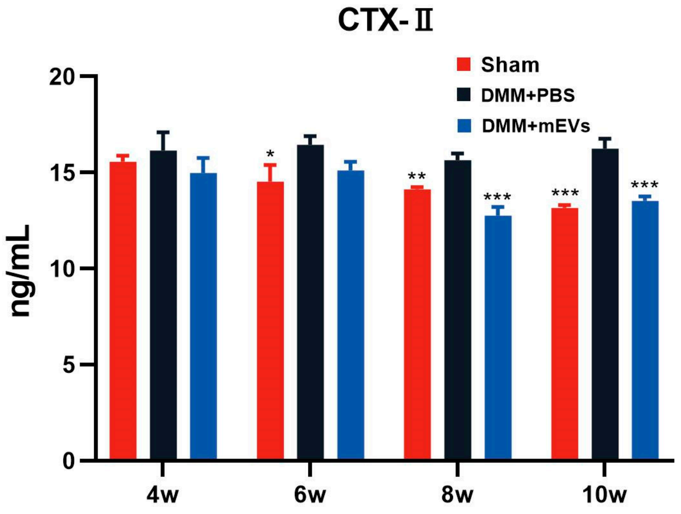

Figure S1 The serum CTX-II levels were determined by ELISA. All data are shown as means ± SD deviation (n = 3). *$p < 0.05$, **$p < 0.01$, ***$p < 0.001$ vs. DMM+PBS group.

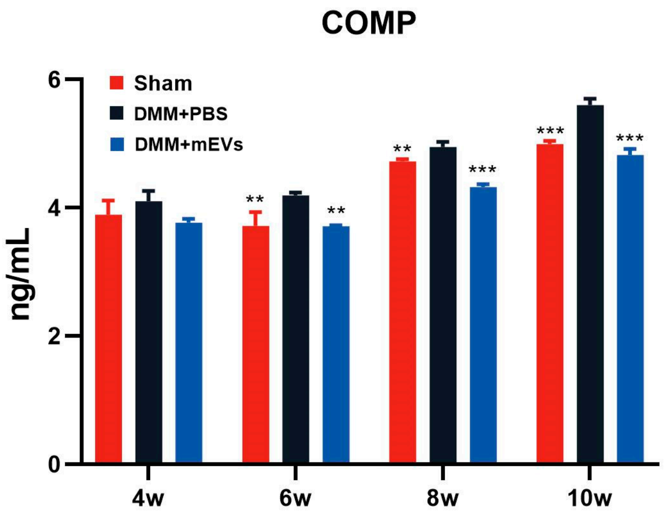

Figure S2 The serum COMP levels were determined by ELISA. All data are shown as means ± SD deviation (n = 3). **$p < 0.01$, ***$p < 0.001$ vs. DMM+PBS group.

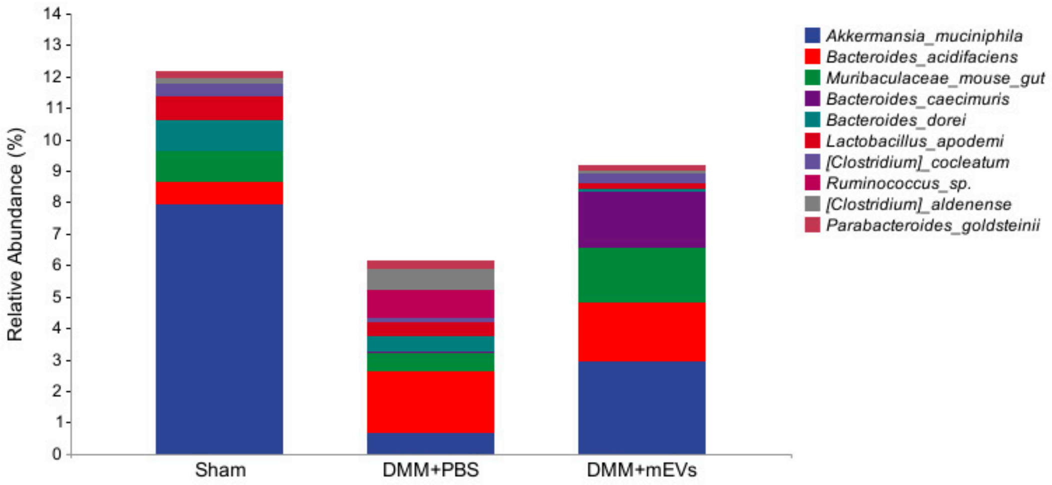

Figure S3 The relative abundance of species in each experimental group.

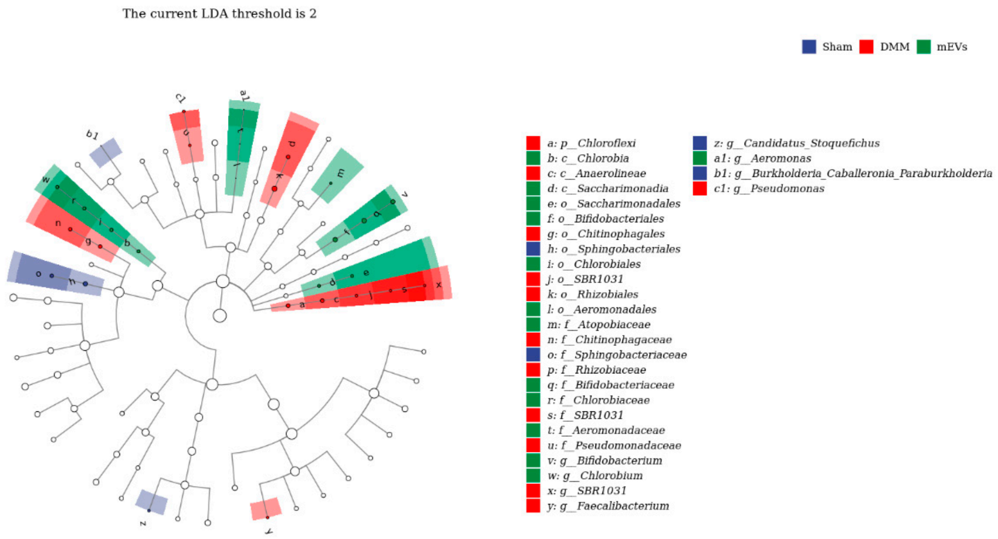

Figure S4 Cladogram based on linear discriminant analysis effect size (LEfSe) analysis showing community composition of the gut microbiota in mice.
